# Cell Tracking Accuracy Measurement Based on Comparison of Acyclic Oriented Graphs

**DOI:** 10.1371/journal.pone.0144959

**Published:** 2015-12-18

**Authors:** Pavel Matula, Martin Maška, Dmitry V. Sorokin, Petr Matula, Carlos Ortiz-de-Solórzano, Michal Kozubek

**Affiliations:** 1 Centre for Biomedical Image Analysis, Faculty of Informatics, Masaryk University, Brno, Czech Republic; 2 Department of Molecular Cytology and Cytometry, Institute of Biophysics, Academy of Sciences of the Czech Republic, Brno, Czech Republic; 3 Cancer Imaging Laboratory, Center for Applied Medical Research, University of Navarra, Pamplona, Spain; Pennsylvania State Hershey College of Medicine, UNITED STATES

## Abstract

Tracking motile cells in time-lapse series is challenging and is required in many biomedical applications. Cell tracks can be mathematically represented as acyclic oriented graphs. Their vertices describe the spatio-temporal locations of individual cells, whereas the edges represent temporal relationships between them. Such a representation maintains the knowledge of all important cellular events within a captured field of view, such as migration, division, death, and transit through the field of view. The increasing number of cell tracking algorithms calls for comparison of their performance. However, the lack of a standardized cell tracking accuracy measure makes the comparison impracticable. This paper defines and evaluates an accuracy measure for objective and systematic benchmarking of cell tracking algorithms. The measure assumes the existence of a ground-truth reference, and assesses how difficult it is to transform a computed graph into the reference one. The difficulty is measured as a weighted sum of the lowest number of graph operations, such as *split*, *delete*, and *add* a vertex and *delete*, *add*, and *alter the semantics* of an edge, needed to make the graphs identical. The measure behavior is extensively analyzed based on the tracking results provided by the participants of the first Cell Tracking Challenge hosted by the 2013 IEEE International Symposium on Biomedical Imaging. We demonstrate the robustness and stability of the measure against small changes in the choice of weights for diverse cell tracking algorithms and fluorescence microscopy datasets. As the measure penalizes all possible errors in the tracking results and is easy to compute, it may especially help developers and analysts to tune their algorithms according to their needs.

## Introduction

The cornerstone of many modern live-cell imaging experiments is the ability to automatically track and analyze the motility of cells in time-lapse microscopy images [1, 2]. Cell tracking is an essential step in understanding a large variety of complex biological processes such as the immune response, embryonic development, or tumorigenesis [3].

Automated cell tracking can be formulated as a problem of identifying and segmenting all desired cell occurrences and describing their temporal relationships in the time-lapse series. Because cells can migrate, undergo division or cell death, collide, or enter and leave the field of view, a cell tracking algorithm suitable for daily practice must reliably address all these events and provide a data structure that thoroughly characterizes the behavior of tracked objects, which could be either whole cells or cell nuclei depending on the application.

State-of-the-art cell tracking approaches can be broadly classified into two categories [4]: *tracking by detection* [5–9] and *tracking by model evolution* [4, 10–13]. The former paradigm generally involves two steps. First, a cell or cell nucleus segmentation algorithm identifies all target objects in the entire time-lapse series separately for each frame. Second, the detected objects are associated between successive frames, typically by optimizing a probabilistic objective function. In contrast, the latter paradigm solves both steps simultaneously, usually using either parametric or implicit active contour models.

Regardless of the particular algorithm used, its tracking results can be mathematically represented using an acyclic oriented graph. The vertices of such a graph correspond to the detected objects while its edges coincide with the temporal relationships between them. Non-dividing objects have one successor at most, whereas those that undergo division have two or even more successors in the case of abnormal division. Cell lineage tracking results represented by an acyclic oriented graph form a forest of trees in the graph theory terminology.

With the increasing number of cell tracking algorithms, there is a natural demand for objective comparisons of their performance. In general, there are two aspects of cell tracking algorithms, which are worth being evaluated: segmentation accuracy and tracking accuracy. The former one characterizes the ability of an algorithm to precisely identify pixels (or voxels) occupied by the objects in the images. It usually leads to the comparison of reference and computed regions based on their overlap or distances between their contours [14, 15]. The tracking accuracy evaluates the ability of an algorithm to correctly detect individual objects of interest and follow them in time.

There are two popular approaches to measuring the tracking accuracy. One approach is based on the ratio of completely reconstructed tracks to the total number of ground-truth tracks [4, 16]. The second computes the ratio of correct temporal relationships within reconstructed tracks to the total number of temporal relationships within ground-truth tracks [16, 17]. Both approaches quantify, at different scales, how well the cell tracking algorithms are able to reconstruct a particular ground-truth reference. However, they neither penalize detecting spurious tracks nor account for division events, which are often evaluated separately [4, 16].

A comprehensive framework for evaluating the performance of the detection and tracking algorithms was established in the field of computer vision [18]. Nevertheless, it targets only topologically stable objects, such as human faces, text boxes, and vehicles. Therefore, it cannot be applied to cell tracking applications because the tracked objects can divide over time or disappear after undergoing cell death. Similarly, another evaluation framework [19], established for comparing the performance of particle tracking methods, does not consider division events, ruling out its ability to evaluate correct cell lineage reconstruction.

In this paper, we propose a tracking accuracy measure that penalizes all possible errors in tracking results and aggregates them into a single value. The measure assesses the difficulty of transforming a computed acyclic oriented graph into a given ground-truth reference. Such difficulty is measured as a weighted sum of the lowest number of graph operations required to make the graphs identical.

The proposed measure can serve not only algorithm developers, but also analysts in order to choose the most suitable algorithm and tune its parameters with respect to all tracking events by optimizing a single criterion. A typical scenario is to create ground truth and evaluate the prospective algorithms on a part of image data, and let the most suitable algorithm run on the rest of it. An alternative way of comparing the performance of algorithms without the need for ground truth has been proposed recently in [20, 21]. However, this approach creates the ranking based on a pairwise comparison of the algorithms and therefore the absolute performance of the algorithms remains unknown.

A prototype of the proposed measure has been continuously used in the individual editions of the Cell Tracking Challenge (http://www.codesolorzano.com/celltrackingchallenge/) being an open and ongoing competition focused on objective and systematic comparison of state-of-the-art cell tracking algorithms [22]. In comparison to [22] where the measure prototype was only sketched out and primarily used as a black-box tool with fixed weights for ranking the algorithms, the contribution of this paper is twofold. First, we provide a rigorous mathematical description of the proposed measure and an extensive study of its behavior, in particular with respect to the choice of weights, based on diverse fluorescence microscopy datasets. It is shown that the proposed measure is robust and stable against small changes in the choice of weights and that the weights can be set to compile rankings strongly correlated with human expert appraisal while reflecting the importance of a particular type of error. Second, a slight modification in the definition of edge-related graph operations, which has no practical impact on the ranking compilation, allows us to formulate the necessary condition for the choice of weights, which guarantees the measure value to be not only the weighted sum of the lowest number of graph operations but also the minimum weighted cost of transforming a computed graph into a given ground-truth reference.

## Materials and Methods

### Proposed cell tracking accuracy measure informally

The main purpose of the proposed measure is to evaluate the ability of cell tracking algorithms to detect all desired objects and follow them in time. Although it does not directly evaluate the accuracy of segmented regions, reliable object detection is a very important factor in this measure as well.

In fact, the measure counts the number of all detection as well as linking errors committed by the algorithm. It counts the number of missed objects (*FN*—false negatives), the number of extra detected objects (*FP*—false positives), and the number of missed splits (required to correctly segment clusters, *NS*). Having those three numbers of errors, we can aggregate them into one number as the weighted sum *w*_*NS*_ *NS* + *w*_*FN*_ *FN* + *w*_*FP*_ *FP* with non-negative weights *w*_*NS*_, *w*_*FN*_, and *w*_*FP*_. The ability of the algorithm to correctly identify temporal relationships between the objects is evaluated by counting the number of errors in object linking. Namely, it counts the number of missing links (*EA*), the number of redundant links (*ED*), and the number of links with the wrong semantics (*EC*). These numbers are again aggregated into one number as the weighted sum *w*_*EA*_ *EA* + *w*_*ED*_ *ED* + *w*_*EC*_ *EC* with non-negative weights *w*_*EA*_, *w*_*ED*_, and *w*_*EC*_. The weights are penalties for individual types of errors and can, for example, reflect the manual effort needed to correct the errors in some particular software.

The number of committed errors can be calculated by counting differences between the ground-truth reference and the computed result where each can be mathematically represented by an acyclic oriented graph. From the computational point of view, a critical part of the proposed measure is the existence of a unique way of pairing reference and computed objects (i.e., graph vertices). To this end, we pair a reference object with a computed one if and only if the latter covers the majority of the former, which guarantees the uniqueness of established pairing and thus straightforward computation without any optimization. Interestingly, such a simple test does not exist for the particle tracking problem, where particles are considered volumeless, making similar evaluation procedure computationally unfeasible.

In the rest of this section, the basic terminology and notation necessary to establish a connection between cell lineage tracking results and acyclic oriented graphs is introduced, and the proposed measure is formally defined.

### Basic terminology and notation

Let *N* be the number of frames in a time-lapse series, *L* be a set of object labels shared among all frames, and ⊥ ∉ *L* be a background label. Let a marker $M_{i}^{t}$ be a set of pixels or voxels of a unique label *i* ∈ *L* in the *t*-th frame, *t* ∈ {0, …, *N* − 1}, related to a particular object. Such markers can be manually created by experts, automatically computed by cell tracking algorithms, established by combining both in an edit-based framework [23], or inherently generated using a simulation toolkit as digital phantoms [24, 25]. A track *θ*_*i*_ is defined as the longest temporal series of markers $M_{i}^{t_{\text{init}}},\cdots ,M_{i}^{t_{\text{end}}}$, 0 ≤ *t*_*init*_ ≤ *t*_*end*_ ≤ *N* − 1, without temporal gaps. When a particular object temporarily disappears from a frame, the corresponding track terminates and a new one with a unique label is established once the object reappears in another frame. Analogously, when a particular object undergoes division, the track of the mother object terminates and new daughter tracks with unique labels are initiated. In either case, the new established tracks are descendants of the terminated tracks. We denote a set of all tracks for the particular time-lapse series with the symbol Θ. To keep the information about relationships between individual tracks, let P:Θ→L∪{⊥} be a parent function defined as

$$\begin{array}{c} P\left(\theta_{i}\right)=\{\begin{array}{cc} j & \;\text{if}\;\theta_{i}\;\text{is}\;a\;\text{descendant}\;\text{of}\;\theta_{j}\;,\\ ⊥ & \;\text{otherwise}\;. \end{array} \end{array}$$ (1)

For simplicity, we also define two other functions, I:Θ→{0,⋯,N-1} and T:Θ→{0,⋯,N-1}, returning zero-based indices of the initial and terminal frames, *t*_*init*_ and *t*_*end*_, for each track. Finally, tracking results for the particular time-lapse series can be expressed as a quadruple (Θ,P,I,T).

**Fig 1:**
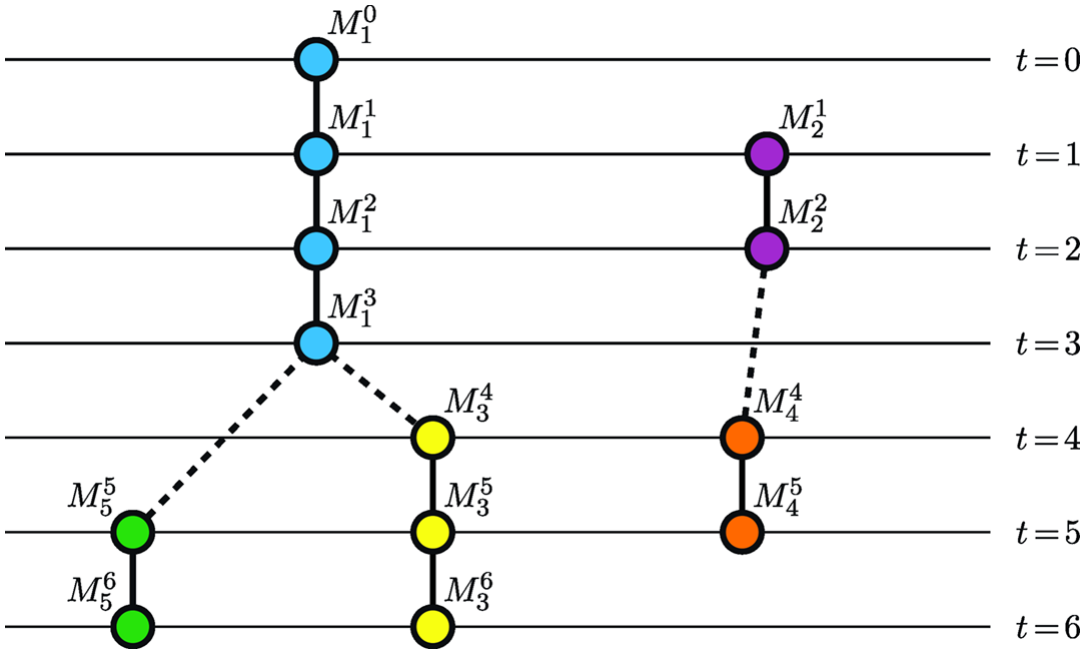
An example of tracking results represented by an acyclic oriented graph. Individual tracks are visualized using different colors. Solid lines correspond to track links, whereas parent links are depicted using dashed lines.

Any quadruple (Θ,P,I,T) can be directly transformed into an acyclic oriented graph *G* = (*V*, *E*) where a set of vertices *V* is composed of all markers present in tracks *θ*_*i*_ ∈ Θ and a set of oriented edges *E* ⊂ *V* × *V* represents temporal relationships between the markers. More precisely, a pair $\left(M_{i}^{t_{1}},M_{j}^{t_{2}}\right)$ is an edge of the graph *G* if and only if either *i* = *j* ∧ *t*_2_ = *t*_1_ + 1 or $i\neq j\wedge t_{1}<t_{2}\wedge T\left(\theta_{i}\right)=t_{1}\wedge I\left(\theta_{j}\right)=t_{2}\wedge P\left(\theta_{j}\right)=i$. In the former case, the edge connects two successive markers within a single track, whereas the terminal marker of *θ*_*i*_ is linked to the initial one of *θ*_*j*_ in the latter case. Hereinafter, we refer to the former edge as *track link* and to the latter one as *parent link*. Over the set of edges *E*, we define a function S:E→{T,P} describing the semantics of an edge *e* ∈ *E* as S(e)=T for track links and S(e)=P for parent links. Note that the orientation of the edges follows the ascending temporal ordering of markers within as well as between tracks, which ensures acyclicity of the graph *G*. An example of the graph *G* is depicted in Fig 1.

### Acyclic oriented graphs matching (*AOGM*) measure

Let *G*_*R*_ = (*V*_*R*_, *E*_*R*_) be a reference graph for a time-lapse series and *G*_*C*_ = (*V*_*C*_, *E*_*C*_) be a computed graph, the accuracy of which we want to evaluate with respect to the reference one. For clarity and brevity, we denote the reference graph vertices as $R_{i}^{t}$, and the computed graph vertices as $C_{j}^{t}$. To determine whether a reference marker $R_{i}^{t}$ was found, we exploit a simple binary test

$$\begin{array}{c} |R_{i}^{t}\cap C_{j}^{t}\left|>0.5\cdot \right|R_{i}^{t}|\;, \end{array}$$ (2)

checking whether a computed marker $C_{j}^{t}$ covers the majority of the reference marker $R_{i}^{t}$. Note that each reference marker can be assigned to one computed marker at most, whereas one computed marker can have multiple reference markers assigned using this detection test. For instance, the latter happens when a cell division was not detected by the algorithm, leaving the daughter cells clustered in a single marker $C_{j}^{t}$. In the case of positive detection test, we write $R_{i}^{t}⋐C_{j}^{t}$ and say that $R_{i}^{t}$ is assigned to $C_{j}^{t}$. Analogously, we consider edges $e_{R}=\left(R_{i}^{t_{1}},R_{j}^{t_{2}}\right)$ and $e_{C}=\left(C_{k}^{t_{1}},C_{l}^{t_{2}}\right)$ to match if and only if the corresponding vertices have positive detection tests (i.e., $e_{R}⋐e_{C}↔R_{i}^{t_{1}}⋐C_{k}^{t_{1}}\wedge R_{j}^{t_{2}}⋐C_{l}^{t_{2}}$).

Based on the detection test, we classify the reference vertices *V*_*R*_ as either true positive $V_{R}^{TP}$ or false negative $V_{R}^{FN}$ in the following way:

- True positives: the correctly detected objects (i.e., reference markers assigned to a computed marker):
  $$\begin{array}{c} V_{R}^{TP}=\left\{R_{i}^{t}\in V_{R}:R_{i}^{t}⋐C_{j}^{t}\;\text{for}\;\text{some}\;C_{j}^{t}\in V_{C}\right\}\;. \end{array}$$ (3)
  We denote the number of true positive vertices as $TP=|V_{R}^{TP}|$.
- False negatives: the missed objects (i.e., reference markers not assigned to any computed marker):
  $$\begin{array}{c} V_{R}^{FN}=\left\{R_{i}^{t}\in V_{R}:R_{i}^{t}⋐C_{j}^{t}\;\text{for}\;\text{no}\;C_{j}^{t}\in V_{C}\right\}\;. \end{array}$$ (4)
  We denote the number of false negative vertices as $FN=|V_{R}^{FN}|$.

Note that each vertex is included in exactly one set and their union contains all reference vertices (i.e., $V_{R}=V_{R}^{FN}\cup V_{R}^{TP}$).

Similarly, we classify the computed vertices *V*_*C*_ as true positive $V_{C}^{TP}$, false positive $V_{C}^{FP}$, or non-split vertices *V* ^*VS*^ in the following way:

- False positives: the extra detected objects (i.e., computed markers without any reference marker assigned):
  $$\begin{array}{c} V_{C}^{FP}=\left\{C_{j}^{t}\in V_{C}:R_{i}^{t}⋐C_{j}^{t}\;\text{for}\;\text{no}\;R_{i}^{t}\in V_{R}\right\}\;. \end{array}$$ (5)
  We denote the number of false positive vertices as $FP=|V_{C}^{FP}|$.
- Non-split vertices: the computed markers with more than one reference marker assigned:
  $$\begin{array}{c} V_{C}^{VS}=\left\{C_{j}^{t}\in V_{C}:R_{k}^{t}⋐C_{j}^{t},R_{l}^{t}⋐C_{j}^{t}\;\text{for}\;\text{some}\;R_{k}^{t},R_{l}^{t}\in V_{R},k\neq l\right\}\;. \end{array}$$ (6)
  We denote the number of non-split vertices as $VS=|V_{C}^{VS}|$. When *m* reference markers (*m* > 1) are assigned to a single computed marker, *m* − 1 *split vertex* operations need to be performed to locally equalize the number of vertices of the reference and computed graphs. These operations decompose the computed marker into *m* non-empty, disjoint markers, such that each of the *m* reference markers is assigned to exactly one of them. The total number of *split vertex* operations can be easily obtained as the difference between the number of true positive reference vertices and the number of the computed graph vertices with a reference marker assigned:
  $$\begin{array}{c} NS=TP-|\{C_{j}^{t}\in V_{C}:R_{i}^{t}⋐C_{j}^{t}\;\text{for}\;\text{some}\;R_{i}^{t}\in V_{R}\}|\;. \end{array}$$ (7)
- True positives: the computed markers with exactly one reference marker assigned:
  $$\begin{array}{c} V_{C}^{TP}=V_{C}\setminus \left(V_{C}^{VS}\cup V_{C}^{FP}\right)\;. \end{array}$$ (8)

Note that each computed vertex is included in exactly one set and their union contains all computed vertices (i.e., $V_{C}=V_{C}^{FP}\cup V_{C}^{TP}\cup V_{C}^{VS}$).

Knowing vertex classification, we define edge-related errors by comparing the reference edges with those in an induced subgraph $\hat{G_{C}}=\left(\hat{V_{C}},\hat{E_{C}}\right)$ of the computed graph *G*_*C*_ by a vertex set $\hat{V_{C}}=V_{C}^{TP}$, which is formed of the uniquely matching computed vertices (i.e., those with exactly one reference vertex assigned) and all their incident edges (i.e., $\hat{E_{C}}=\left\{\left(C_{i}^{t_{1}},C_{j}^{t_{2}}\right)\in E_{C}:C_{i}^{t_{1}},C_{j}^{t_{2}}\in V_{C}^{TP}\right\}$).

First, we define the set of redundant edges in the computed graph. These are the induced subgraph edges without any counterpart in the reference graph:

$$\begin{array}{c} E_{C}^{FP}=\{\left(C_{i}^{t_{1}},C_{j}^{t_{2}}\right)\in \hat{E_{C}}:R_{k}^{t_{1}}⋐C_{i}^{t_{1}},R_{l}^{t_{2}}⋐C_{j}^{t_{2}}\;\text{for}\;\text{some}\;\\ R_{k}^{t_{1}},R_{l}^{t_{2}}\in V_{R},\left(R_{k}^{t_{1}},R_{l}^{t_{2}}\right)\notin E_{R}\}\;. \end{array}$$ (9)

We denote the number of redundant edges as $ED=|E_{C}^{FP}|$. Note that the computed edges attached to a false positive vertex or non-split vertex are not included because they are inherently removed together with deleting the false positive vertices or with splitting the non-split vertices.

Analogously, we define the set of missing edges in the computed graph. These are the reference graph edges without any counterpart in the induced subgraph:

$$\begin{array}{c} E_{R}^{FN}=\left\{e_{R}\in E_{R}:e_{R}⋐e_{C}\;\text{for}\;\text{no}\;e_{C}\in \hat{E_{C}}\right\}\;. \end{array}$$ (10)

We denote the number of missing edges as $EA=|E_{C}^{FN}|$.

Finally, we define the set of edges with wrong semantics in the computed graph. These are the matching edges between the reference graph and the induced subgraph, which differ in semantics:

$$\begin{array}{c} E_{C}^{CS}=\left\{e_{C}\in \hat{E_{C}}:e_{R}⋐e_{C}\;\text{for}\;\text{some}\;e_{R}\in E_{R},S\left(e_{R}\right)\neq S\left(e_{C}\right)\right\}\;. \end{array}$$ (11)

We denote the number of such edges as $EC=|E_{C}^{CS}|$.

The transformation of any computed graph into the reference one involves the following procedure. First, one can build a binary matrix with |*V*_*R*_| columns and |*V*_*C*_| rows containing the results of the detection test given by Eq (2) for each pair of vertices $\left(R_{i}^{t},C_{j}^{t}\right)$, $R_{i}^{t}\in V_{R}$, $C_{j}^{t}\in V_{C}$. Due to the majority overlap criterion, each reference vertex is assigned to at most one computed vertex. Therefore, either none or only one match can appear in each column, resulting in either false negative or true positive classification of the respective reference vertices. Similarly, the matrix rows without any match reveal false positive vertices, and those with multiple matches correspond to the computed vertices that need to be split. Note that the number of matches in each such row decremented by one gives the number of splits that must be executed on the particular non-split vertex to locally equalize the number of vertices in both graphs. Because the reference as well as computed markers are spatially localized, the vertex matching is unique. In total, *NS* *split vertex* operations, *FN* *add vertex* operations, and *FP* *delete vertex* operations need to be performed to have the vertex sets of both graphs matching. Subsequently, we remove redundant edges, add missing ones, and finally correct those with wrong semantics. These operations are also unique because any of them cannot be replaced by a reasonable combination of the others. This requires *ED* *delete edge* operations, *EA* *add edge* operations, and *EC* *alter the edge semantics* operations, respectively.

The weighted sum of the executed operations is considered as the cost of transforming the computed graph into the reference one (*AOGM* measure):

$$\begin{array}{c} AOGM=w_{NS}NS+w_{FN}FN+w_{FP}FP+w_{ED}ED+w_{EA}EA+w_{EC}EC\;. \end{array}$$ (12)

**Fig 2:**
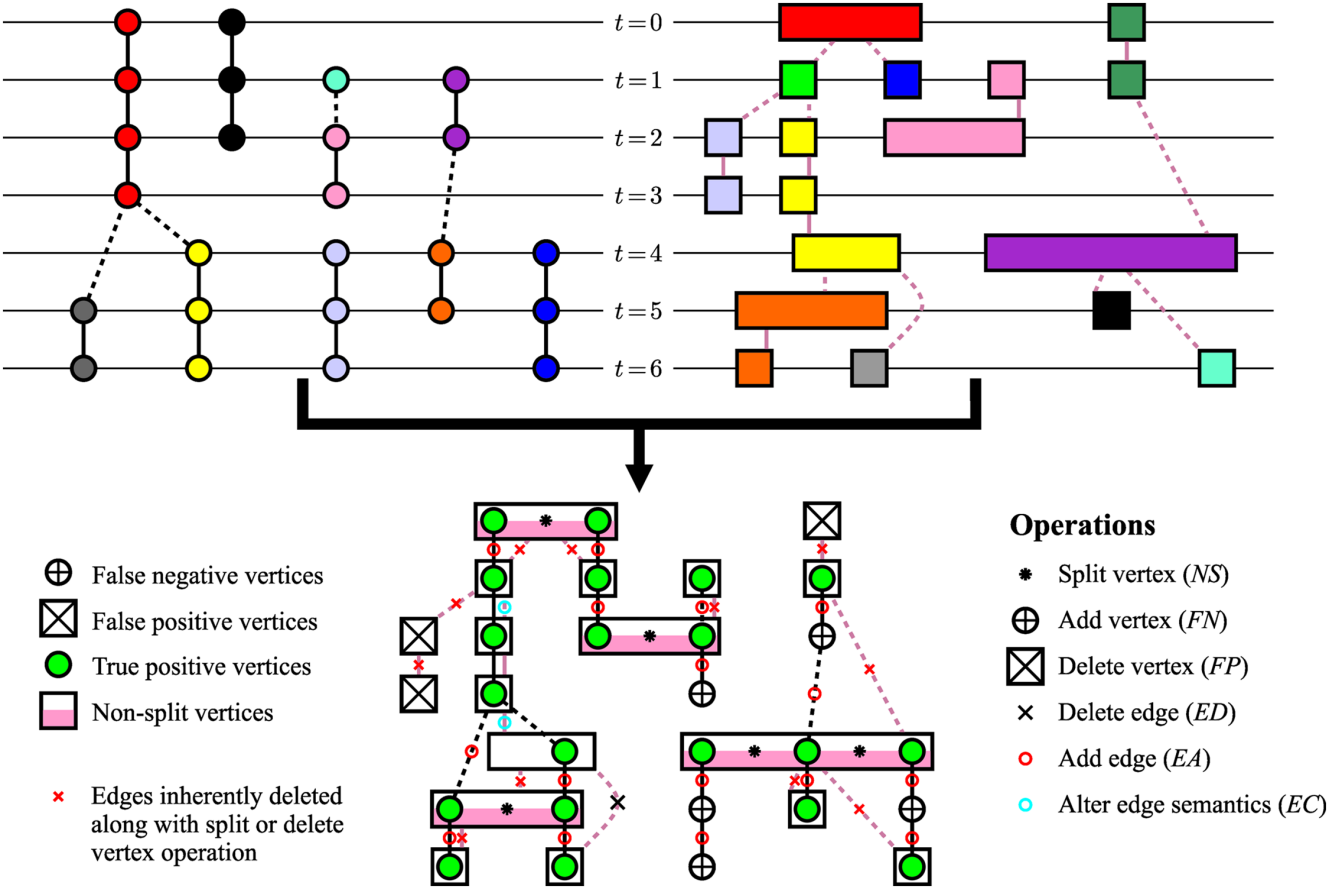
An example of calculating the *AOGM* measure for a reference graph (upper left) formed of circular vertices and black edges and a computed graph (upper right) formed of rectangular vertices and dark pink edges. In both graphs, the vertical axis represents the temporal domain and the horizontal axis represents the spatial domain (i.e., each vertex has a certain spatial extent). The *AOGM* measure is the weighted sum of the following quantities (bottom): the number of splits (*NS* = 5, black asterisks in pink-white rectangles) computed as the difference between the number of true positive vertices (20 green circles) and the number of white and pink-white rectangles containing at least one green circle (15), the number of false negative vertices (*FN* = 5, white circles with the black plus sign), the number of false positive vertices (*FP* = 3, white rectangles with the black cross), the number of redundant edges (*ED* = 1, black cross), the number of missing edges (*EA* = 16, small red circles), and finally the number of edges with wrong semantics (*EC* = 2, small blue circles).

With the assumption of non-negative weights along with at least one weight being positive, the *AOGM* measure is bounded below by zero. Its value is equal to zero when a computed graph is identical to the ground-truth reference or when it contains only errors penalized with a zero weight. The *AOGM* value increases, theoretically to infinity, with the increasing complexity of the transformation that converts the computed graph into the reference one, where complexity is judged with respect to operations penalized with non-zero weights. The higher the *AOGM* value is, the worse output an algorithm has provided and the worse its ranking is. An example of calculating the *AOGM* measure is illustrated in Fig 2.

The ability of an algorithm to detect all important objects can be measured using the *AOGM* measure when keeping only vertex-related weights positive (i.e., *w*_*NS*_, *w*_*FN*_, *w*_*FP*_ > 0; *w*_*ED*_ = *w*_*EA*_ = *w*_*EC*_ = 0). We further refer to such variant of the *AOGM* measure as *AOGM-D*. Analogously, when keeping only the edge-related weights positive (i.e., *w*_*NS*_ = *w*_*FN*_ = *w*_*FP*_ = 0; *w*_*ED*_, *w*_*EA*_, *w*_*EC*_ > 0), the *AOGM* measure evaluates the ability of an algorithm to follow objects in time (i.e., its association skills). We further refer to such variant of the *AOGM* measure as *AOGM-A*.

## Results and Discussion

In this section, we first discuss the minimality of the proposed measure, describe testing data used for the experimental evaluation, and present their properties in terms of tracking errors. Next, we study how sensitive the *AOGM* measure is to the choice of weights, and how its behavior coincides with human expert appraisal. Finally, we discuss the size of necessary ground truth.

### Measure minimality

The computation of the *AOGM* measure is a deterministic procedure, which always returns a single value derived from the number of differences between the computed and reference graphs. Because we can, thanks to the detection test given by Eq (2), uniquely match the graphs, the set of errors is also unique and can be easily determined. The allowed graph operations to correct the errors are *add*, *delete*, and *split* a vertex and *add*, *delete*, and *alter the semantics* of an edge. All but the *split vertex* operation are independent in a sense that they cannot be substituted by a reasonable combination of the others and each operation directly corrects one error. A non-split vertex with *m* reference vertices assigned could also be corrected using the following sequence of operations: delete the non-split vertex and add *m* new vertices. If the cost of this sequence is higher than the cost of *m* − 1 *split vertex* operations, the *AOGM* value is equal to the minimum cost necessary to transform the computed graph into the reference one. We formulate the necessary condition, which guarantees the *AOGM* value to be not only the weighted sum of the lowest number of graph operations but also the minimum weighted sum, as

$$\begin{array}{c} w_{NS}\left(m^{⋆}-1\right)\leq w_{FP}+w_{FN}m^{⋆} \end{array}$$ (13)

where *m* ^⋆^ is the maximum number of reference vertices assigned to a single non-split vertex among all non-split vertices. For practical purposes, the minimality condition can be weakened to *w*_*NS*_ ≤ *w*_*FN*_ to be independent of the input graphs.

Note that the measure prototype used in the first Cell Tracking Challenge calculated the edge-related errors over the entire computed graph and therefore, some of the erroneous edges attached to non-split vertices were inherently corrected along with the vertex splitting while the others were not. This complicated the measure minimality reasoning because the cost of correcting a single non-split vertex may have also involved the penalties for a variable number of *delete edge* and *alter the edge semantics* operations on the left side of Eq (13) and for a variable number of *add edge* operations on the right side of Eq (13), and therefore we slightly modified the measure definition. By calculating the edge-related errors over the induced subgraph only, the correction of vertex-related errors is clearly separated from the edge-related ones, allowing minimality condition to be formulated only using the vertex-related weights. In general, the proposed measure penalizes the splitting errors slightly higher than the measure used in the first Cell Tracking Challenge, in particular due to a slight increase in the number of *add edge* operations, being in average approximately 1.88 per non-split vertex, caused by inherent deleting of all edges attached to non-split vertices during their splittings. However, such increase does not have any practical impact on the compiled rankings and they remain unchanged for all algorithms and datasets used in the first Cell Tracking Challenge regardless of the way how the edges attached to non-split vertices are handled.

### Testing data

We experimentally validated the proposed measure using the results of four cell tracking algorithms (COM-US, HEID-GE, KTH-SE, and PRAG-CZ as named in [22]) that participated in the first Cell Tracking Challenge. These algorithms provided complete tracking results for the entire competition dataset repository [22], which allowed us to study the *AOGM* measure behavior under distinct scenarios involving, in particular, various cell phenotypes (i.e., shape, density, and motion model) and acquisition configurations (i.e., imaging system, image data dimensionality, and time step). The basic properties of the competition datasets are listed in Table 1. As our primary interest lies in analyzing the *AOGM* measure behavior with respect to the choice of weights in Eq (12), rather than in compiling a ranking of the competing algorithms and in discussing their strengths and weaknesses, details about the algorithms are omitted. We further refer to them as A1, A2, A3, and A4 in a random but fixed order.

**Table 1:** Basic properties of the competition repository of diverse fluorescence microscopy datasets analyzed in this work.

Dataset	Objects of interest	Imaging system	Dim.	Time step [min]	No. of frames	No. of series
**1**	Rat mesenchymal stem cells	PerkinElmer Ultraview ERS	2D	20	48	2
**2**	H157 human lung cancer cells	PerkinElmer Ultraview ERS	3D	2	60	2
**3**	MDA231 breast carcinoma cells	Olympus FluoView F1000	3D	80	12	2
**4**	Nuclei of embryonic stem cells	Leica TCS SP5	2D	5	92	2
**5**	Nuclei of HeLa cells	Olympus IX81	2D	30	92	2
**6**	Nuclei of CHO cells	Zeiss LSM 510	3D	9.5	92	2
**7**	Nuclei of HL60 cells	Simulation toolkit	2D	28.8–57.6	56–100	6
**8**	Nuclei of HL60 cells	Simulation toolkit	3D	28.8–57.6	56–100	6

In this work, two types of ground-truth references were used, depending on the origin of a particular dataset, referred as real and synthetic datasets. They were adopted without any modification from [22]. For the real datasets, three experts were requested to place a quintessential marker inside every object and establish temporal relationships between the markers to provide an acyclic oriented graph for each time-lapse series. Because it is widely documented in the literature that humans commit errors when performing manual tracking [26, 27], the reference graphs were created based on majority voting to reduce human errors. For the synthetic datasets, the reference graphs were inherently generated by a simulation toolkit [24, 25] along with the time-lapse series to be analyzed.

### Distribution of tracking errors

**Fig 3:**
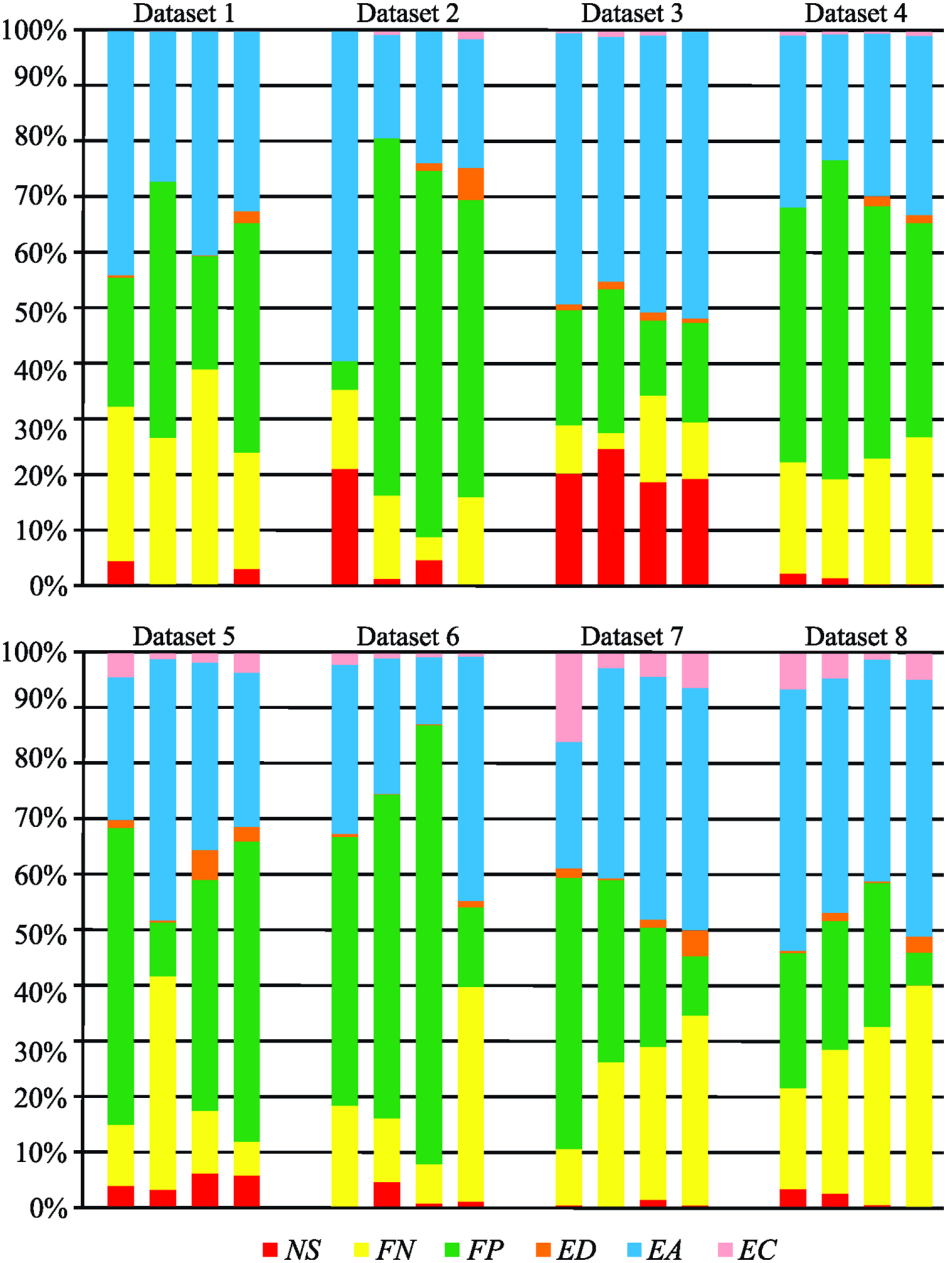
The distribution of errors committed by the four tested algorithms for individual datasets. Each group of four horizontal bars corresponds to the tested algorithms depicted in the same order for each dataset.

We focused on the statistical analysis of errors in tracking results produced by the four tested algorithms to reveal the distribution of errors under distinct scenarios displayed in the competition datasets (Table 1). The average number and standard deviation of particular errors, normalized either per ground-truth vertex or per ground-truth edge to facilitate the comparison between datasets with different numbers of objects, are listed in Table 2. The distribution of errors that the algorithms made in particular datasets is shown in Fig 3. The measured values revealed three key observations. First, from the computed standard deviations of errors, it can be observed that the tested algorithms made different types of errors in each particular dataset. Second, there is a preponderance of the *add edge* operations over the *add vertex* ones because correcting a false negative vertex requires adding at least one edge to integrate the vertex into a particular track, except when a whole track is formed of a single vertex. Furthermore, missing edges may need to be added also due to inherent deletion of all edges attached to non-split vertices. Third, the predominance of a specific error seems to be dataset-dependent: the false negative detection predominates in Datasets 5 and 8, the incorrectly clustered objects in Dataset 3, and the false positive detection in the remaining datasets.

**Table 2:** The average number and standard deviation of errors in tracking results normalized either per ground-truth vertex or per ground-truth edge depending on a particular error type. The second and third columns list the aggregated numbers of vertices and edges in the ground-truth references per dataset.

Dataset	⋃_*i*_|*V*_*R*_*i*__|	⋃_*i*_|*E*_*R*_*i*__|	*μ*_*NS*_ ± *σ*_*NS*_	*μ*_*FN*_ ± *σ*_*FN*_	*μ*_*FP*_ ± *σ*_*FP*_	*μ*_*ED*_ ± *σ*_*ED*_	*μ*_*EA*_ ± *σ*_*EA*_	*μ*_*EC*_ ± *σ*_*EC*_
**1**	915	885	0.017 ± 0.018	0.510 ± 0.267	0.651 ± 0.494	0.011 ± 0.015	0.607 ± 0.229	0.001 ± 0.001
**2**	490	478	0.012 ± 0.012	0.057 ± 0.069	0.232 ± 0.300	0.003 ± 0.003	0.097 ± 0.076	0.003 ± 0.003
**3**	846	767	0.159 ± 0.072	0.064 ± 0.030	0.155 ± 0.085	0.010 ± 0.005	0.400 ± 0.102	0.006 ± 0.005
**4**	5432	5364	0.009 ± 0.011	0.142 ± 0.125	0.385 ± 0.445	0.003 ± 0.003	0.185 ± 0.160	0.002 ± 0.002
**5**	29374	29136	0.012 ± 0.011	0.095 ± 0.148	0.059 ± 0.019	0.004 ± 0.003	0.132 ± 0.174	0.004 ± 0.002
**6**	1993	1968	0.010 ± 0.014	0.059 ± 0.027	0.222 ± 0.167	0.001 ± 0.001	0.097 ± 0.052	0.002 ± 0.001
**7**	11741	11617	0.001 ± 0.001	0.035 ± 0.031	0.039 ± 0.040	0.001 ± 0.001	0.052 ± 0.045	0.005 ± 0.002
**8**	12286	12164	0.002 ± 0.001	0.059 ± 0.059	0.045 ± 0.049	0.002 ± 0.001	0.080 ± 0.070	0.004 ± 0.001
**Total**	63077	62379	0.028 ± 0.053	0.128 ± 0.181	0.223 ± 0.312	0.004 ± 0.007	0.206 ± 0.218	0.003 ± 0.003

### Sensitivity to the choice of weights

We investigated the behavior of the *AOGM* measure with respect to the choice of weights in Eq (12). As a reference weight configuration, we adopted the weights used in the first Cell Tracking Challenge, which reflected the effort needed to correct a particular error manually: *w*_*NS*_ = 5 for *vertex splitting*, *w*_*FN*_ = 10 for *vertex adding*, *w*_*FP*_ = 1 for *vertex deleting*, *w*_*ED*_ = 1 for *edge deleting*, *w*_*EA*_ = 1.5 for *edge adding*, and *w*_*EC*_ = 1 for *altering the edge semantics*. Note that such weight configuration satisfies the minimality condition given by Eq (13) for any *m* ^⋆^.

**Fig 4:**
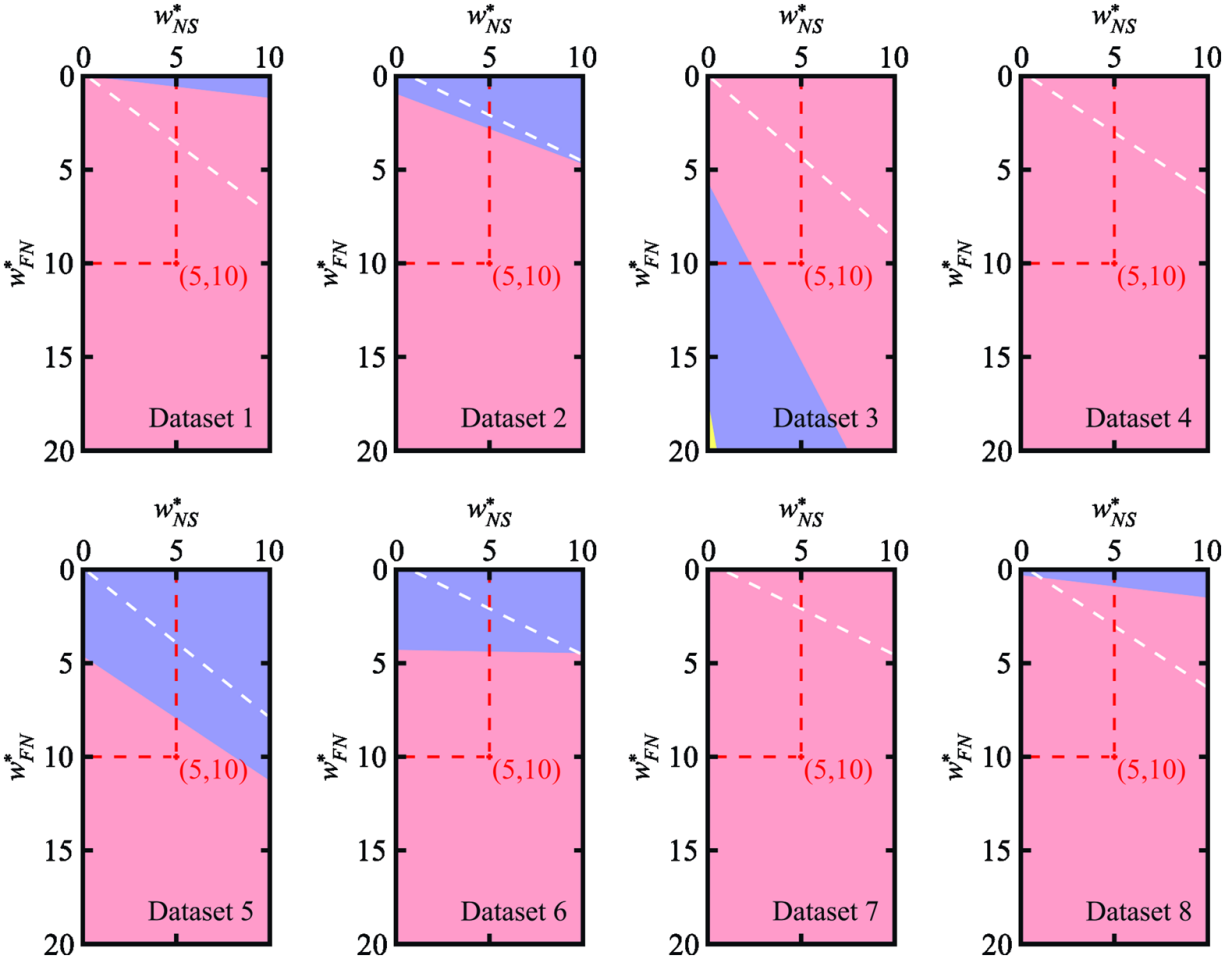
Pairwise disjoint sectors of all possible configurations $\left(w_{NS}^{*},w_{FN}^{*}\right)$ within the rectangular domain 〈0, 10〉 × 〈0, 20〉, which simultaneously preserve a particular ranking for each individual dataset. The red crosses mark the reference configuration $\left(w_{NS}^{*},w_{FN}^{*}\right)=\left(5,10\right)$ used for compiling the reference rankings. The upper-right corners outlined by the white lines consist of the configurations, which break the minimality condition given by Eq (13). The colors encode the numbers of transpositions in the particular rankings compared to the reference ones. No transposition occurs in the pink regions, one transposition occurs in the blue regions, and two transpositions occur in the tiny yellow region.

We wanted to know how a change in the setting of weights would have affected the reference rankings of the four tested algorithms in terms of tracking ability. To this end, we computed sectors in weight space leading to the same rankings. The analysis was carried out with respect to the pair of the two most influential weights, *w*_*NS*_ and *w*_*FN*_. The other weights were fixed at their original values, serving as normalization factors. By solving systems of linear inequalities in two unknown weights, $w_{NS}^{*}$ and $w_{FN}^{*}$, within the rectangular domain 〈0, 10〉 × 〈0, 20〉, we studied how many transpositions in the obtained rankings occurred, compared to those compiled for the reference configuration $\left(w_{NS}^{*},w_{FN}^{*}\right)=\left(5,10\right)$. The domain was chosen to range from 0 up to a double magnitude of the reference weight in each axis, being centered at the reference weight configuration. Due to the linearity of Eq (12), the solutions of these systems form polygonal sectors, each consisting of the configurations leading to the same ranking. The results for all datasets are shown in Fig 4. The borderlines between the sectors correspond to the configurations, for which at least two algorithms gained the same *AOGM* value. Almost all tested configurations led to no more than one transposition in the reference rankings, and even in the half of the datasets, we observed practically no changes in the reference rankings. We also carried out a similar study for another two pairs of weights, namely (*w*_*FP*_, *w*_*FN*_) and (*w*_*EA*_, *w*_*FN*_), which turned out to be the weights of most frequent errors across all the datasets. The number of sectors and their relative areas are listed in Table 3. Note that in all of the cases the largest sector was formed of the configurations leading to the reference ranking. The sectors with no more than one transposition occupied the whole studied domain in all but three cases. However, in these three cases, they covered more than 99% of the domain.

**Table 3:** The number of sectors with different rankings and their relative areas within the rectangular domain centered at the reference weight configuration for individual datasets. The results for three different pairs of weights, (*w*_*NS*_, *w*_*FN*_), (*w*_*FP*_, *w*_*FN*_), and (*w*_*EA*_, *w*_*FN*_), are presented. The columns 0, 1, and 2 contain the relative areas of the sectors with the particular number of transpositions given by the column label.

Pair	Dataset	No. of sectors	0	1	2
(*w*_*NS*_, *w*_*FN*_)	1	2	97.15	2.85	0.00
	2	2	85.94	14.06	0.00
	3	3	73.08	26.57	0.35
	4	1	100.00	0.00	0.00
	5	2	60.25	39.75	0.00
	6	2	78.11	21.89	0.00
	7	1	100.00	0.00	0.00
	8	2	95.54	4.46	0.00
(*w*_*FP*_, *w*_*FN*_)	1	2	97.09	2.91	0.00
	2	2	85.94	14.06	0.00
	3	2	76.04	23.96	0.00
	4	1	100.00	0.00	0.00
	5	2	60.25	39.75	0.00
	6	4	74.91	24.64	0.45
	7	2	99.97	0.03	0.00
	8	2	95.54	4.46	0.00
(*w*_*EA*_, *w*_*FN*_)	1	2	97.01	2.99	0.00
	2	2	85.94	14.06	0.00
	3	2	76.04	23.96	0.00
	4	2	99.69	0.31	0.00
	5	2	60.25	39.75	0.00
	6	3	78.11	21.22	0.67
	7	2	99.43	0.57	0.00
	8	2	95.54	4.46	0.00

Based on the information depicted in Fig 4 and Table 3, it can be concluded that rankings of the tested algorithms compiled using the *AOGM* measure are highly robust against small changes in the choice of weights. This demonstrates that the reference configuration $\left(w_{NS}^{*},w_{FN}^{*}\right)=\left(5,10\right)$ compiles the predominant rankings of the tested algorithms for diverse datasets (Table 1) because it belongs to the sectors of the largest area (the pink sectors in Fig 4, and the column 0 in Table 3).

### Comparison with human expert appraisal

To validate the *AOGM* measure against human expert appraisal, we asked three independent human experts to rank the algorithms according to two criteria, which were formulated as (1) “the ability of the algorithm to detect all important objects”, shortly detection performance, and (2) “the ability of the algorithm to follow objects in time”, shortly association performance. We did not provide any special instructions on how the experts were supposed to rank the algorithms and we let their decision completely on their opinion. In particular, we did not provide them with the ground truth and did not require any quantification, because it would tend toward the manual counting of errors in the results, which is basically what the *AOGM* measure does automatically, provided the ground truth exists. The experts allocated points to the algorithms from 0 to 3 (3 means the best) for detection and association separately and the total score in each category was computed by summing up the points. The overall score was obtained by pure addition of the total score in each category.

The rankings compiled using *AOGM-D* and *AOGM-A* variants of the *AOGM* measure were compared with human expert rankings on all the six real 2-D sequences. The results are summarized in Tables 4, 5 and 6. They indicate high correlation, with the Kendall rank correlation coefficient *τ* ranging from 0.78 up to 0.83, between the rankings on the basis of the proposed method and human expert opinions. The perfect agreement occurred for three analyzed sequences (Dataset 1/Sequence 1, Dataset 1/Sequence 2, and Dataset 4/Sequence 2).

**Table 4:** The detection ranking comparison of the four tested algorithms (A1–A4) with human expert appraisal on real 2-D dataset sequences using *AOGM-D* with weights *w*_*NS*_ = 5, *w*_*FN*_ = 10, *w*_*FP*_ = 1; *w*_*ED*_ = *w*_*EA*_ = *w*_*EC*_ = 0. The black bullets mark the matches in the rankings.

Dataset/Sequence	*AOGM-D*				Ranking				Expert points				Ranking			
	A1	A2	A3	A4	A1	A2	A3	A4	A1	A2	A3	A4	A1	A2	A3	A4
**1/1**	1415	6376	4977	2879	1	4	3	2	9	0	4	5	•1	•4	•3	•2
**1/2**	420	2599	1748	963	1	4	3	2	8	0	3	7	•1	•4	•3	•2
**4/1**	2101	10125	2998	3870	1	4	2	3	4	0	9	5	3	•4	1	2
**4/2**	1311	16342	1589	1806	1	4	2	3	9	0	6	3	•1	•4	•2	•3
**5/1**	2281	57113	4785	1587	2	4	3	1	9	0	4	5	1	•4	•3	2
**5/2**	1813	52985	3244	2095	1	4	3	2	8	0	6	4	•1	•4	2	3

**Table 5:** The association ranking comparison of the four tested algorithms (A1–A4) with human expert appraisal on real 2-D dataset sequences using *AOGM-A* with weights *w*_*NS*_ = *w*_*FN*_ = *w*_*FP*_ = 0; *w*_*ED*_ = 1, *w*_*EA*_ = 1.5, *w*_*EC*_ = 1. The black bullets mark the matches in the rankings.

Dataset/Sequence	*AOGM-A*				Ranking				Expert points				Ranking			
	A1	A2	A3	A4	A1	A2	A3	A4	A1	A2	A3	A4	A1	A2	A3	A4
**1/1**	274.5	889.0	738.5	551.0	1	4	3	2	9	1	2	6	•1	•4	•3	•2
**1/2**	98.5	280.0	257.0	177.5	1	4	3	2	9	0	4	5	•1	•4	•3	•2
**4/1**	364.5	1629.0	511.0	612.0	1	4	2	3	5	0	9	4	2	•4	1	•3
**4/2**	255.0	2105.5	258.5	319.5	1	4	2	3	8	0	7	3	•1	•4	•2	•3
**5/1**	479.5	9517.5	1141.0	452.5	2	4	3	1	9	0	4	5	1	•4	•3	2
**5/2**	499.0	9703.5	1334.0	753.5	1	4	3	2	8	0	6	4	•1	•4	2	3

**Table 6:** The overall ranking comparison of the four tested algorithms (A1–A4) with human expert appraisal in terms of detection on real 2-D dataset sequences using *AOGM* with weights *w*_*NS*_ = 5, *w*_*FN*_ = 10, *w*_*FP*_ = 1; *w*_*ED*_ = 1, *w*_*EA*_ = 1.5, *w*_*EC*_ = 1. The overall AOGM values and expert points were obtained by pure addition of the respective quantities for the detection (Table 4) and association (Table 5) parts. The black bullets mark the matches in the rankings.

Dataset/Sequence	*AOGM*				Ranking				Expert points				Ranking			
	A1	A2	A3	A4	A1	A2	A3	A4	A1	A2	A3	A4	A1	A2	A3	A4
**1/1**	1689.5	7265.0	5715.5	3430.0	1	4	3	2	18	1	6	11	•1	•4	•3	•2
**1/2**	518.5	2879.0	2005.0	1140.5	1	4	3	2	17	0	7	12	•1	•4	•3	•2
**4/1**	2465.5	11754.0	3509.0	4482.0	1	4	2	3	9	0	18	9	2	•4	1	•3
**4/2**	1566.0	18447.5	1847.5	2125.5	1	4	2	3	17	0	13	6	•1	•4	•2	•3
**5/1**	2760.5	66630.5	5926.0	2039.5	2	4	3	1	18	0	8	10	1	•4	•3	2
**5/2**	2312.0	62688.5	4578.0	2848.5	1	4	3	2	16	0	12	8	•1	•4	2	3

The difference in the rankings for Dataset 4/Sequence 1 exists because the human experts preponderated the existence of non-split objects and false positive objects over the existence of false negative objects during the evaluation process. Tables 7 and 8 show that the *AOGM* measure could have compiled the same ranking as that compiled by the human experts if their preference on the type of errors was reflected in the weights. The same observation holds for Dataset 5/Sequence 1. Similarly, the swap in *AOGM-A* for Dataset 4/Sequence 1 exists because *AOGM-A* puts more emphasis on the missing edges rather than on their semantics. If the weights were accordingly altered, we could have obtained the human expert ranking. In Dataset 5/Sequence 2, A3 was slightly worse than A4 with respect to all types of errors. However, the human experts ranked this algorithm higher. We suppose it is because the human expert comparison was sometimes very difficult and subjective, especially if the algorithms behaved similarly, and it was impracticable to manually compute the precise number of errors due to image data complexity (e.g., Dataset 5 contains hundreds of cells per frame).

**Table 7:** The ranking comparison of the four tested algorithms (A1–A4) with human expert appraisal in terms of detection on real 2-D dataset sequences using *AOGM-D* with weights *w*_*NS*_ = 10, *w*_*FN*_ = 1, *w*_*FP*_ = 10; *w*_*ED*_ = *w*_*EA*_ = *w*_*EC*_ = 0. The black bullets mark the matches in the rankings.

Dataset/Sequence	*AOGM-D*				Ranking				Expert points				Ranking			
	A1	A2	A3	A4	A1	A2	A3	A4	A1	A2	A3	A4	A1	A2	A3	A4
**1/1**	1354	5548	2448	5273	1	4	2	3	9	0	4	5	•1	•4	3	2
**1/2**	335	8467	1541	1352	1	4	3	2	8	0	3	7	•1	•4	•3	•2
**4/1**	4867	29954	4339	4743	3	4	1	2	4	0	9	5	•3	•4	•1	•2
**4/2**	1759	36279	3711	2873	1	4	3	2	9	0	6	3	•1	•4	2	3
**5/1**	2926	20456	9519	5035	1	4	3	2	9	0	4	5	•1	•4	•3	•2
**5/2**	10100	24655	11619	10381	1	4	3	2	8	0	6	4	•1	•4	2	3

**Table 8:** The overall ranking comparison of the four tested algorithms (A1–A4) with human expert appraisal on real 2-D dataset sequences using *AOGM* with weights *w*_*NS*_ = 10, *w*_*FN*_ = 1, *w*_*FP*_ = 10; *w*_*ED*_ = 1, *w*_*EA*_ = 1.5, *w*_*EC*_ = 1. The overall AOGM values and expert points were obtained by pure addition of the respective quantities for the detection (Table 7) and association (Table 5) parts. The black bullets mark the matches in the rankings.

Dataset/Sequence	*AOGM*				Ranking				Expert points				Ranking			
	A1	A2	A3	A4	A1	A2	A3	A4	A1	A2	A3	A4	A1	A2	A3	A4
**1/1**	1628.5	6437.0	3186.5	5824.0	1	4	2	3	18	1	6	11	•1	•4	3	2
**1/2**	433.5	8747.0	1798.0	1529.5	1	4	3	2	17	0	7	12	•1	•4	•3	•2
**4/1**	5231.5	31583.0	4850.0	5355.0	2	4	1	3	9	0	18	9	•2	•4	•1	•3
**4/2**	2014.0	38384.5	3969.5	3192.5	1	4	3	2	17	0	13	6	•1	•4	2	3
**5/1**	3405.5	29973.5	10660.0	5487.5	1	4	3	2	18	0	8	10	•1	•4	•3	•2
**5/2**	10599.0	34358.5	12953.0	11134.5	1	4	3	2	16	0	12	8	•1	•4	2	3

The experiments demonstrate that *AOGM* measure can compile expected ranking, being strongly correlated with human expert one, provided the weights reflect the importance of a particular type of error.

### Size of ground truth

Typical cell tracking experiments produce hundreds to thousands of 2-D or 3-D images. A decision on what algorithm to use and how to optimally set its parameters is often not an easy task.

A widely adopted approach is to run several algorithms with different parameter settings and visually compare their results. The size of image data often makes such evaluation practicable on its limited subset only, although being tricky, especially for 3-D experiments, and subjective due to inter-operator variability and a graphical user interface used.

An alternative approach is to create ground truth for a part of image data and use the *AOGM* measure for objective and accurate evaluation of the algorithms. In general, the creation of ground truth is laborious, although the amount of work can be reduced by adopting an edit-based framework [23]. Nevertheless, such effort pays off soon when multiple algorithms need to be evaluated and their parameters optimally tuned.

**Fig 5:**
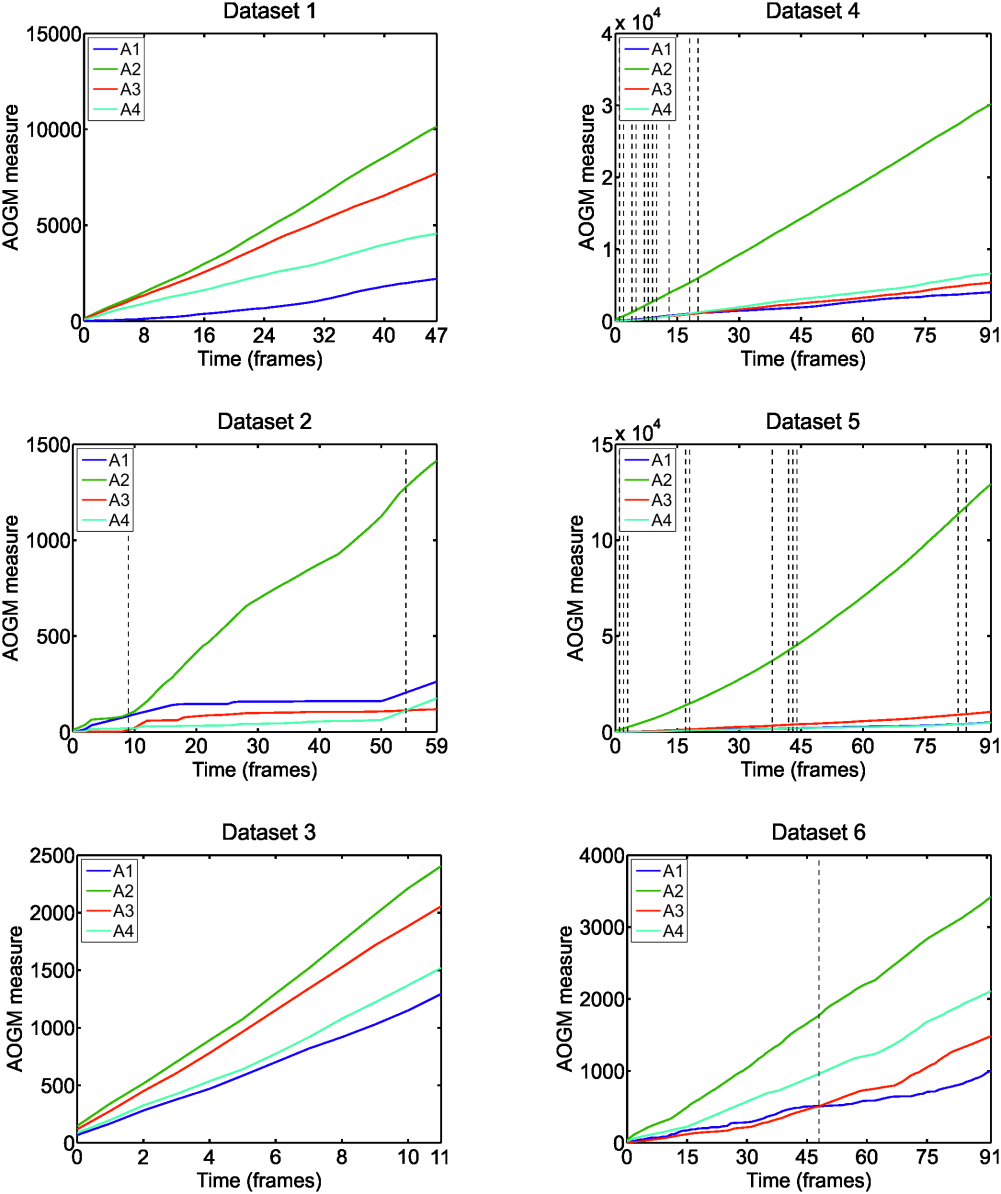
The temporal evolution of the *AOGM* measure, averaged over the two sequences per each real dataset, for the four tested algorithms (A1–A4). The dashed vertical lines indicate the frames at which the algorithm ranking compiled using the *AOGM* measure has changed.

However, any general recommendation on the size of ground truth to get unbiased evaluation of the algorithm performance can be hardly stated. It depends on the complexity of image data and particular application. Instead, we computed the temporal evolution of the *AOGM* measure for each algorithm and each real dataset (Fig 5). It can be observed that the number of frames after which the rankings of the tested algorithms stabilized varied across the datasets. It can also be observed often a linear increase in the *AOGM* value, indicating the tested algorithms committed approximately the same number of errors over time.

## Conclusions

In this paper, the *AOGM* measure, an accuracy measure for objective and systematic comparison of cell tracking algorithms that are capable of providing segmentation of individual cells rather than simplifying them as single-point objects, has been defined and analyzed. Treating tracking results as an acyclic oriented graph, the proposed measure assesses how difficult it is to transform a computed graph into a given ground-truth graph. The cost of such a transformation is defined as the weighted sum of the lowest number of graph operations needed to make the graphs identical. The behavior of the *AOGM* measure was analyzed on tracking results provided by four state-of-the-art cell tracking algorithms that participated in the first Cell Tracking Challenge. The performed analyses verified its robustness and stability against small changes in the choice of weights for diverse fluorescence microscopy datasets.

As the weights chosen in Eq (12) are not biologically motivated, they reflect the effort needed for performing a particular graph operation, the *AOGM* measure is application-independent. A different choice of weights can serve purposes other than objectively and systematically comparing the performance of multiple cell tracking algorithms as presented in this paper. For example, the *AOGM* measure might also be useful to tune the parameters of each single module involved in a cell tracking algorithm under development for a specific dataset. Its *AOGM-D* variant allows one to determine the optimal parameters of a cell detection module. Furthermore, setting the weight *w*_*NS*_ to 1 and the others to 0 allows one to optimize a cluster separation module.

In addition to being robust, stable, and flexible, the *AOGM* measure is also universal. The measure penalizes all possible errors in tracking results, not concentrating on only a single, often application-limited aspect of cell tracking as the existing approaches do [4, 16, 17]. It can be applied to datasets with various characteristics, even nearly degenerated cases such as those showing no division or containing only a single cell. Furthermore, it can be used for evaluating the performance of any cell tracking algorithm irrespective of its nature because it evaluates its final output. Therefore, developers can use the *AOGM* measure to easily compare the performance of a cell tracking algorithm under development to that of existing algorithms, whereas analysts can use it to determine the optimal parameters of a chosen algorithm for a dataset to be analyzed. The software for computing the *AOGM* measure is made publicly available at http://cbia.fi.muni.cz/aogm/ or as a supplementary material S1 Software, free of charge for noncommercial and research purposes.

## Supporting Information

S1 Software AOGMMeasure. This package contains a routine for computing the AOGM measure.
(ZIP)

S1 File Supporting Information File. This supporting information file contains all relevant data used for generating the results described in the manuscript.
(XLSX)
