# Supplementary material for: Cell Tracking Accuracy Measurement Based on Comparison of Acyclic Oriented Graphs
Source: PLoS One. 2015 Dec 18;10(12):e0144959. doi: 10.1371/journal.pone.0144959 (PMC4686175; doi:10.1371/journal.pone.0144959)
Supplement: S1 Software — This package contains a routine for computing the AOGM measure. (ZIP) [file pone.0144959.s001.zip › README.pdf]

AOGMMeasure

This package contains a routine for computing the *AOGM* measure. The routine, purely written in C++, is released as a 32-bit standalone console application for the machines running Windows 7 or later. The routine was dynamically built using the Microsoft Visual Studio C++ 2015 compiler. Thus, please make sure that the Microsoft Visual Studio 2015 Redistributable (x86) package has been installed on your machine before running the routine. Without this package installed, the routine will not run properly, producing no output. It may even complain about a missing msvcp140.dll file.

The routine itself requires eight input parameters to be specified:

- dir_gt – A path to the directory with a reference graph,
- dir_res – A path to the directory with a computed graph,
- w_ns – A penalty for the *split vertex* operation,
- w_fn – A penalty for the *add vertex* operation,
- w_fp – A penalty for the *delete vertex* operation,
- w_ed – A penalty for the *delete edge* operation,
- w_ea – A penalty for the *add add* operation,
- w_ec – A penalty for the *alter the edge semantics* operation.

After specifying the parameters, the routine returns the *AOGM* value on the standard output and creates a log file AOGM_log.txt in the directory with the computed graph. Note that the routine requires both graphs to be saved in the format used in the Cell Tracking Challenge.

To verify proper functionality of the routine, the provided testing directory ("testing_data") can be used. It contains a reference graph ("testing_data\GT") and a computed graph ("testing_data\RES"). They correspond to the graphs depicted in Fig. 2 in the manuscript. The *AOGM* value for the weight configuration (w_ns, w_fn, w_fp, w_ed, w_ea, w_ec) = (5, 10, 1, 1, 1.5, 1) is 105. Such output can be obtained by executing the command:

AOGMMeasure.exe testing_data\GT testing_data\RES 5 10 1 1 1.5 1.

The routine is provided free of charge for noncommerical and research purposes. Its use for any other purpose is generally possible, but solely with the explicit permission of the authors. In case of any questions, please do not hesitate to contact us at xmaska@fi.muni.cz or pam@fi.muni.cz.
