# Supplementary figures and images for: Cell Tracking Accuracy Measurement Based on Comparison of Acyclic Oriented Graphs
Source: PLoS One. 2015 Dec 18;10(12):e0144959. doi: 10.1371/journal.pone.0144959 (PMC4686175; doi:10.1371/journal.pone.0144959)

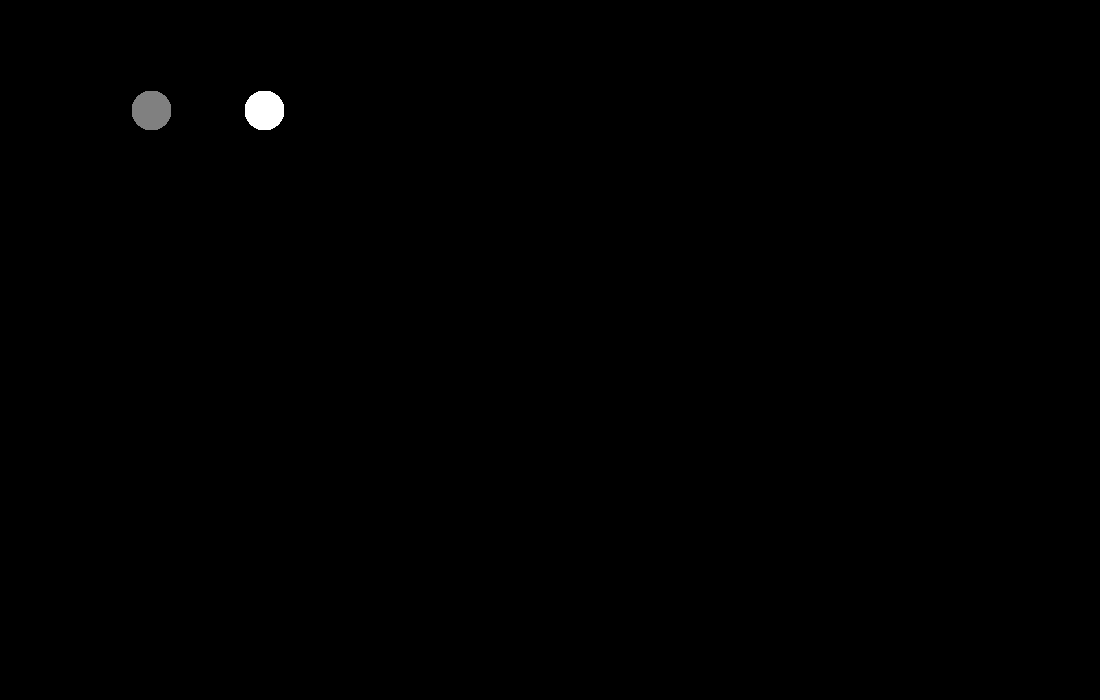

Supplement: S1 Software — This package contains a routine for computing the AOGM measure. (ZIP) [file pone.0144959.s001.zip › testing_data/GT/man_track000.tif]

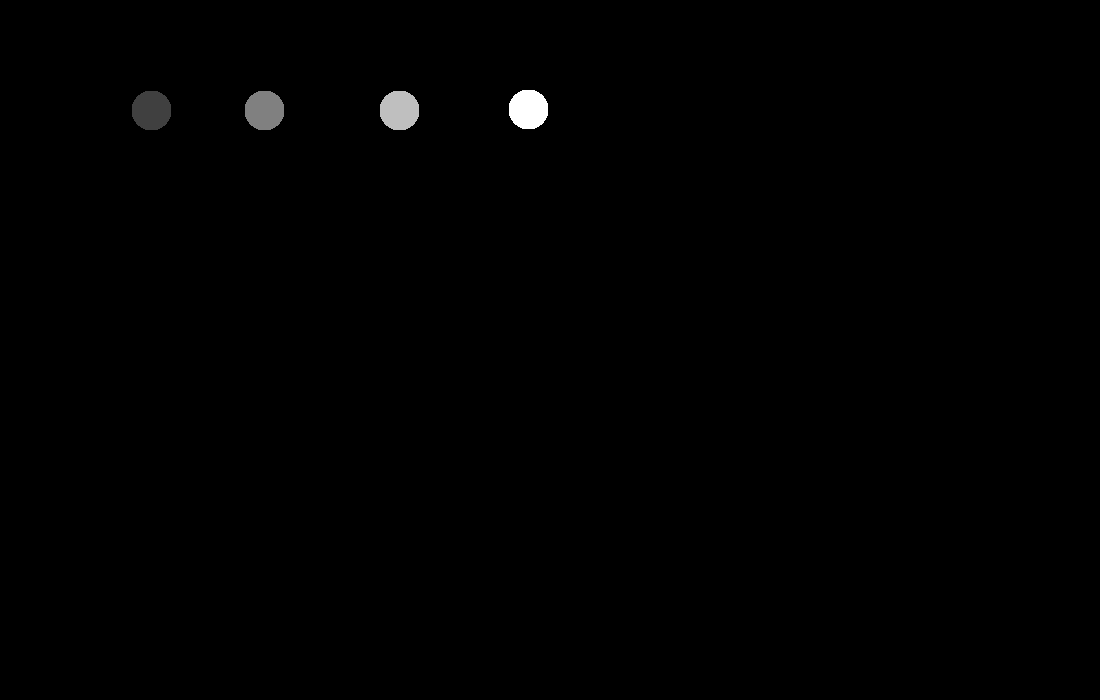

Supplement: S1 Software — This package contains a routine for computing the AOGM measure. (ZIP) [file pone.0144959.s001.zip › testing_data/GT/man_track001.tif]

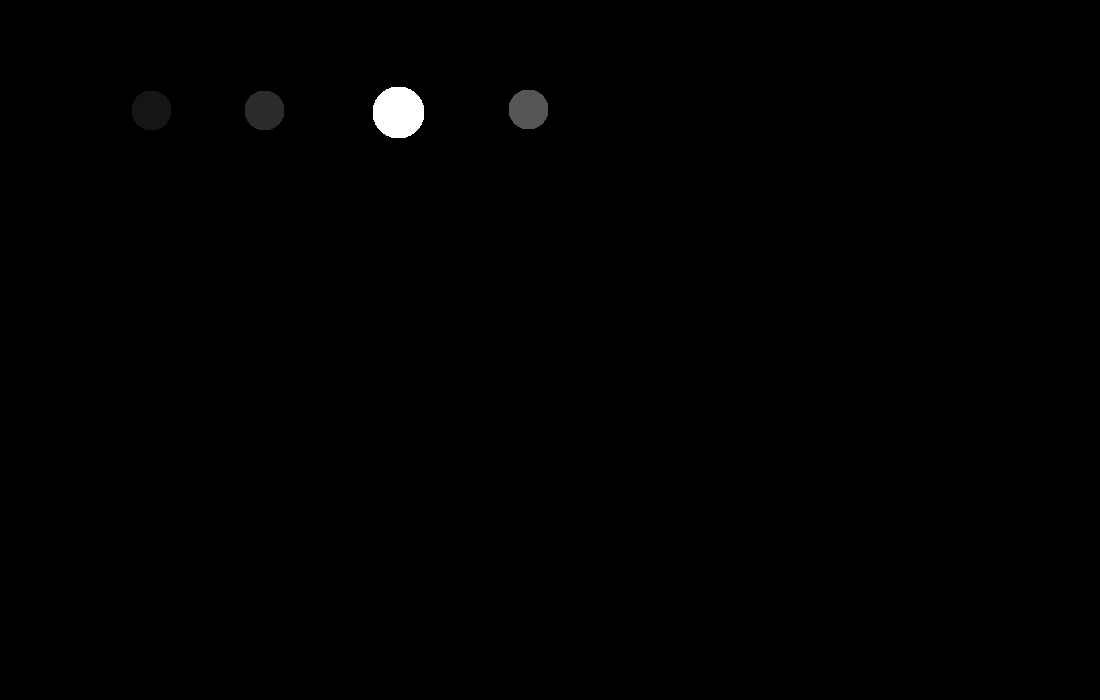

Supplement: S1 Software — This package contains a routine for computing the AOGM measure. (ZIP) [file pone.0144959.s001.zip › testing_data/GT/man_track002.tif]

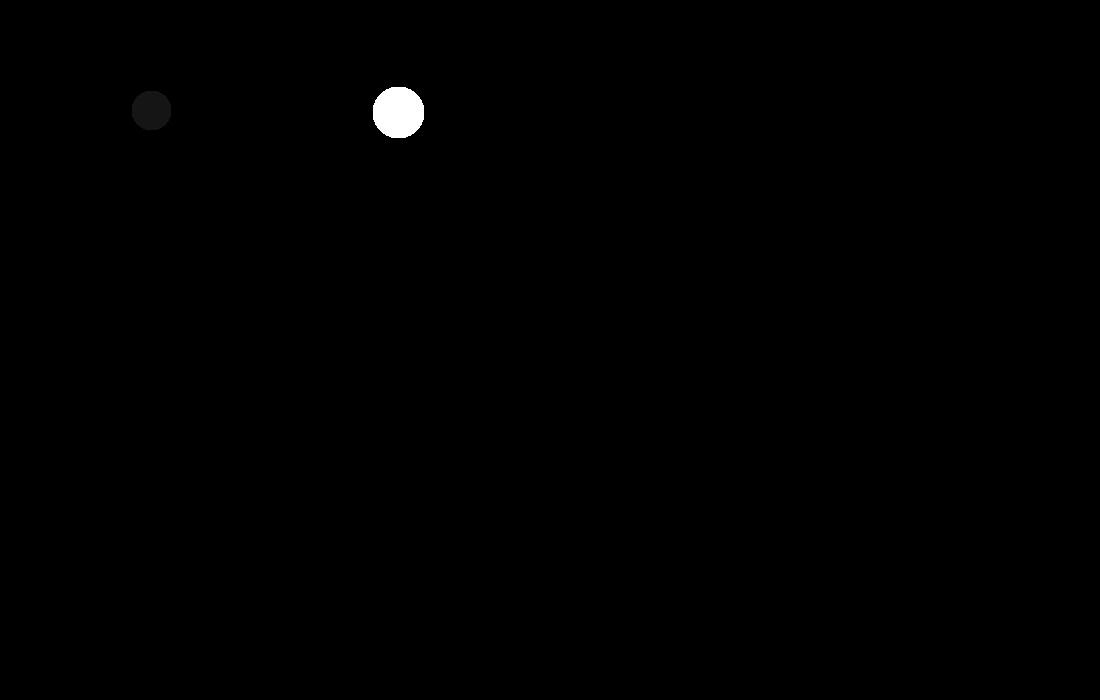

Supplement: S1 Software — This package contains a routine for computing the AOGM measure. (ZIP) [file pone.0144959.s001.zip › testing_data/GT/man_track003.tif]

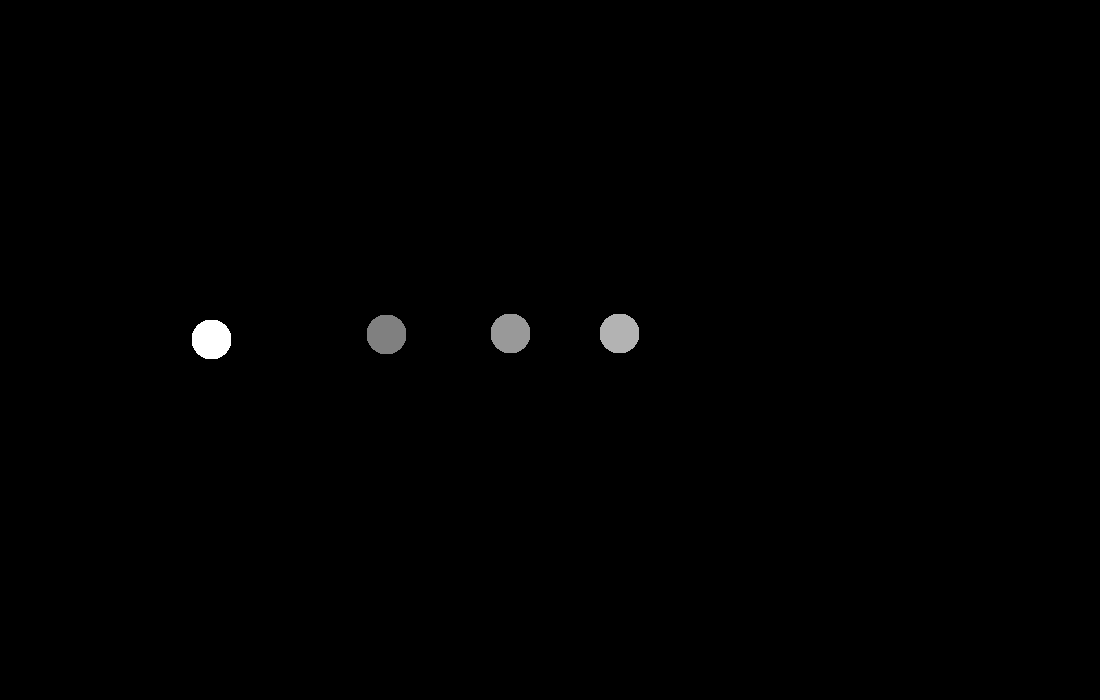

Supplement: S1 Software — This package contains a routine for computing the AOGM measure. (ZIP) [file pone.0144959.s001.zip › testing_data/GT/man_track004.tif]

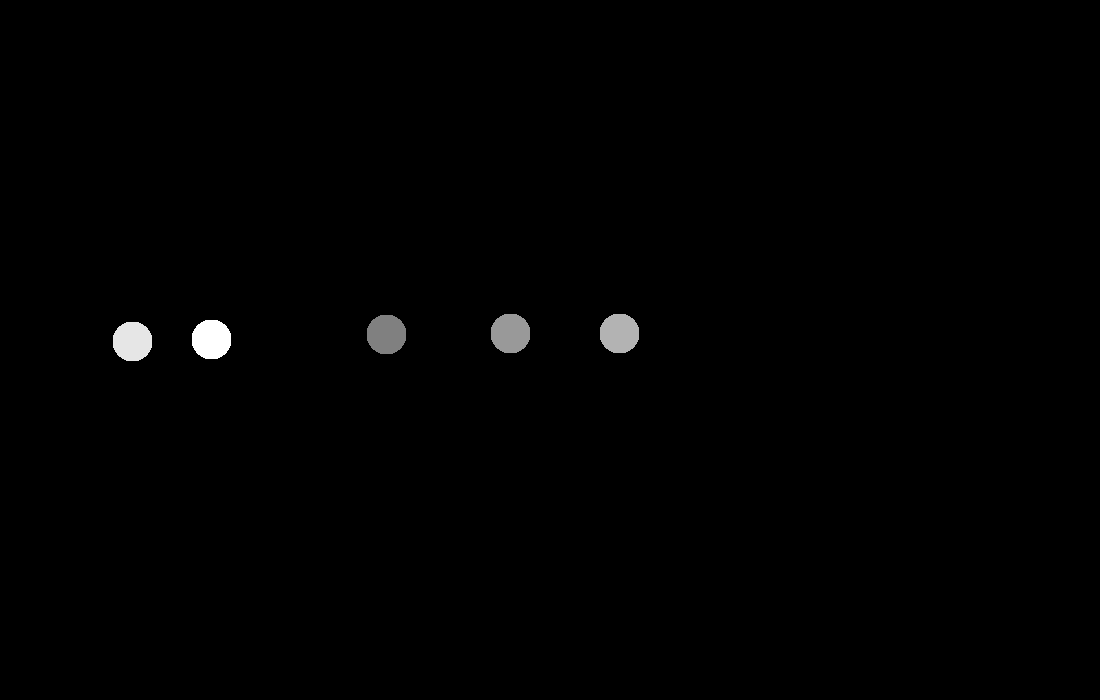

Supplement: S1 Software — This package contains a routine for computing the AOGM measure. (ZIP) [file pone.0144959.s001.zip › testing_data/GT/man_track005.tif]

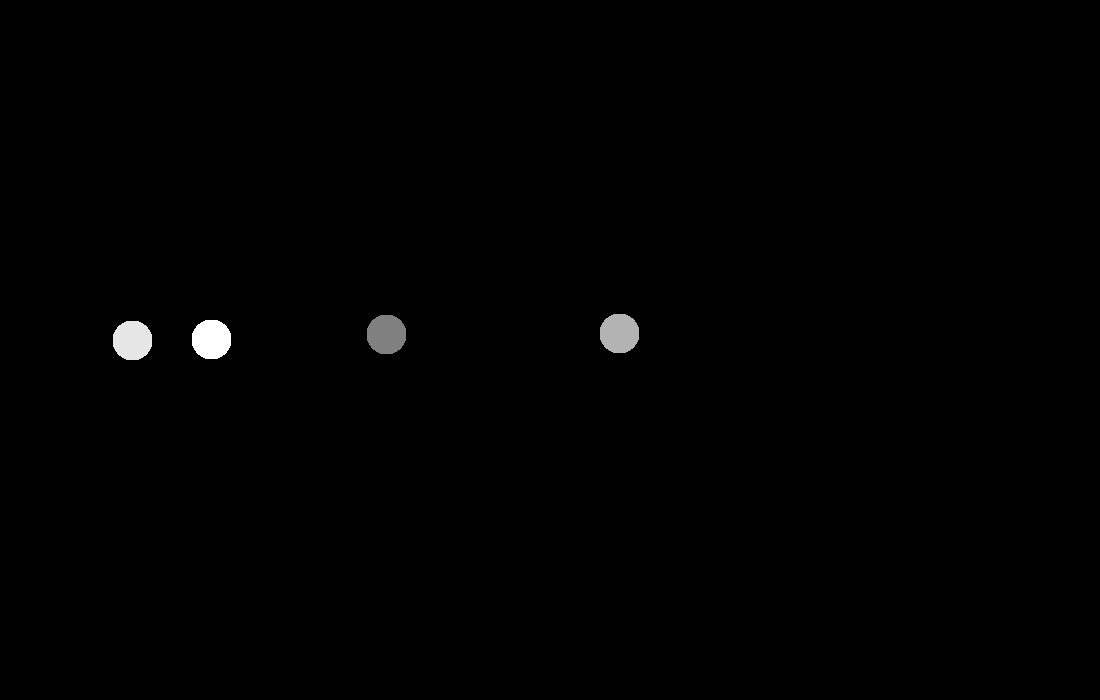

Supplement: S1 Software — This package contains a routine for computing the AOGM measure. (ZIP) [file pone.0144959.s001.zip › testing_data/GT/man_track006.tif]

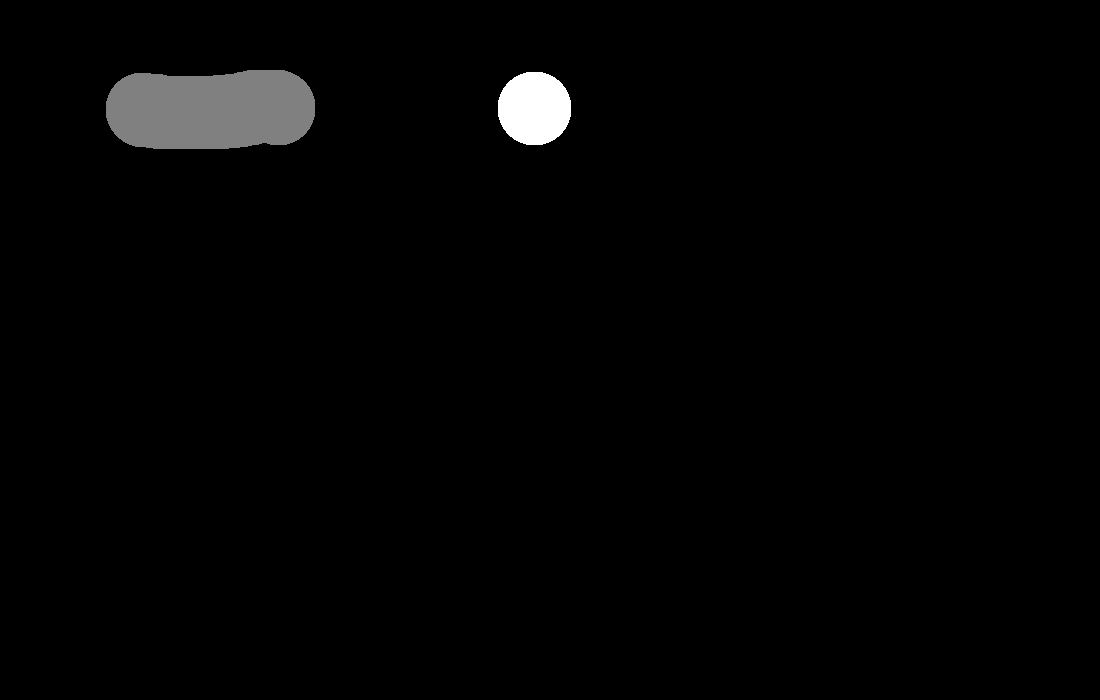

Supplement: S1 Software — This package contains a routine for computing the AOGM measure. (ZIP) [file pone.0144959.s001.zip › testing_data/RES/mask000.tif]

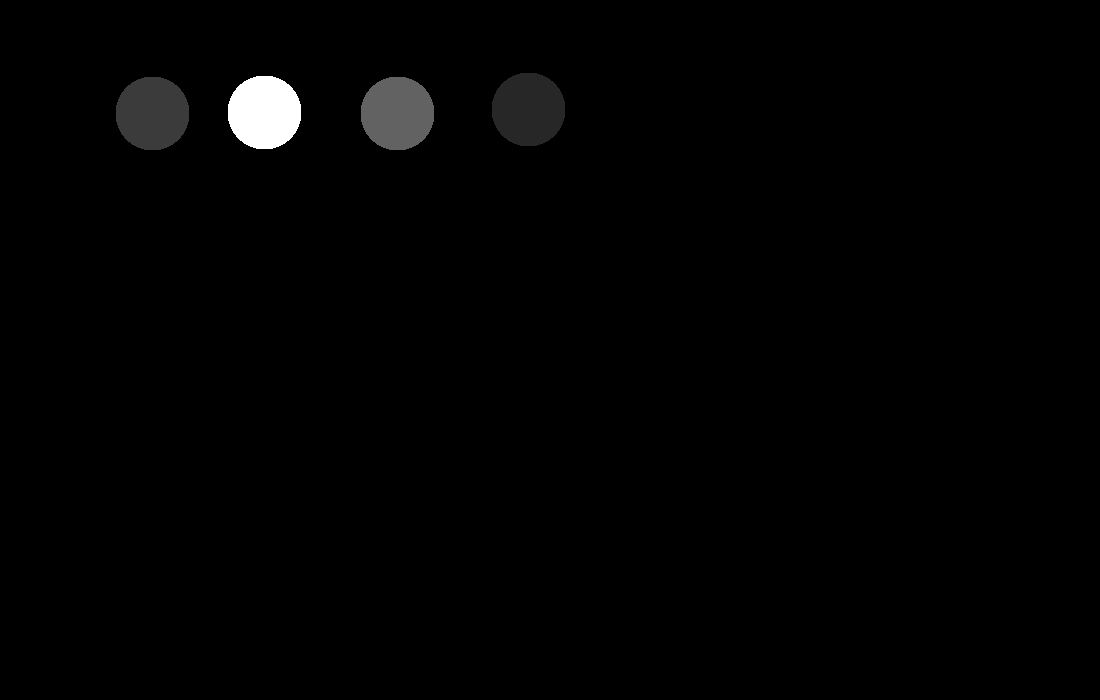

Supplement: S1 Software — This package contains a routine for computing the AOGM measure. (ZIP) [file pone.0144959.s001.zip › testing_data/RES/mask001.tif]

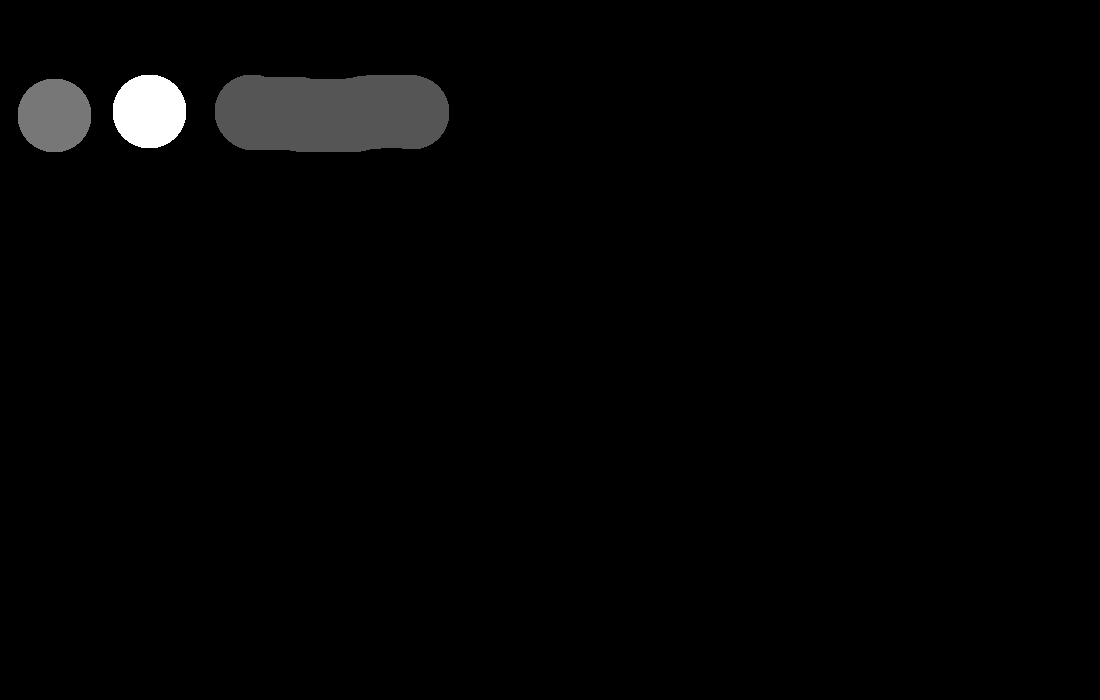

Supplement: S1 Software — This package contains a routine for computing the AOGM measure. (ZIP) [file pone.0144959.s001.zip › testing_data/RES/mask002.tif]

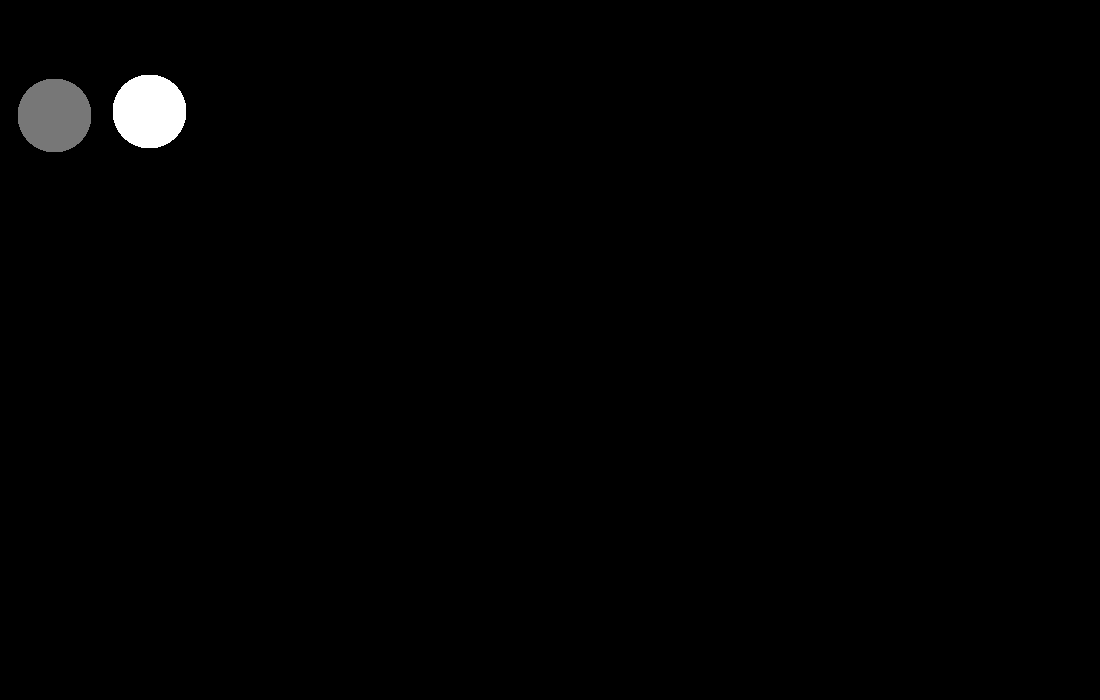

Supplement: S1 Software — This package contains a routine for computing the AOGM measure. (ZIP) [file pone.0144959.s001.zip › testing_data/RES/mask003.tif]

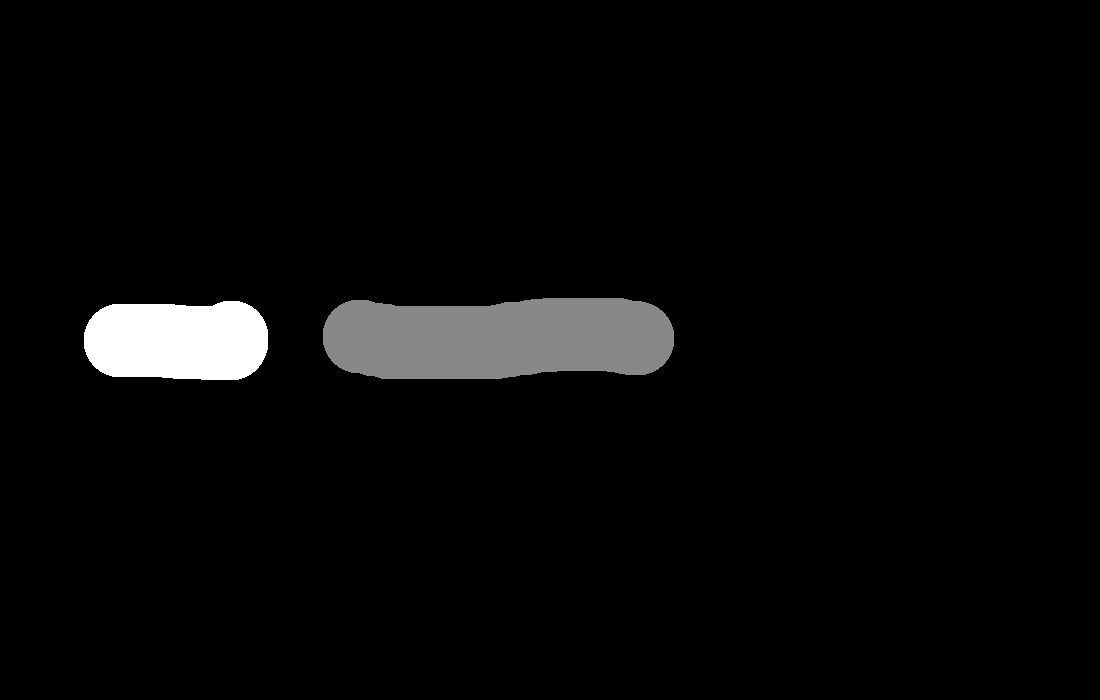

Supplement: S1 Software — This package contains a routine for computing the AOGM measure. (ZIP) [file pone.0144959.s001.zip › testing_data/RES/mask004.tif]

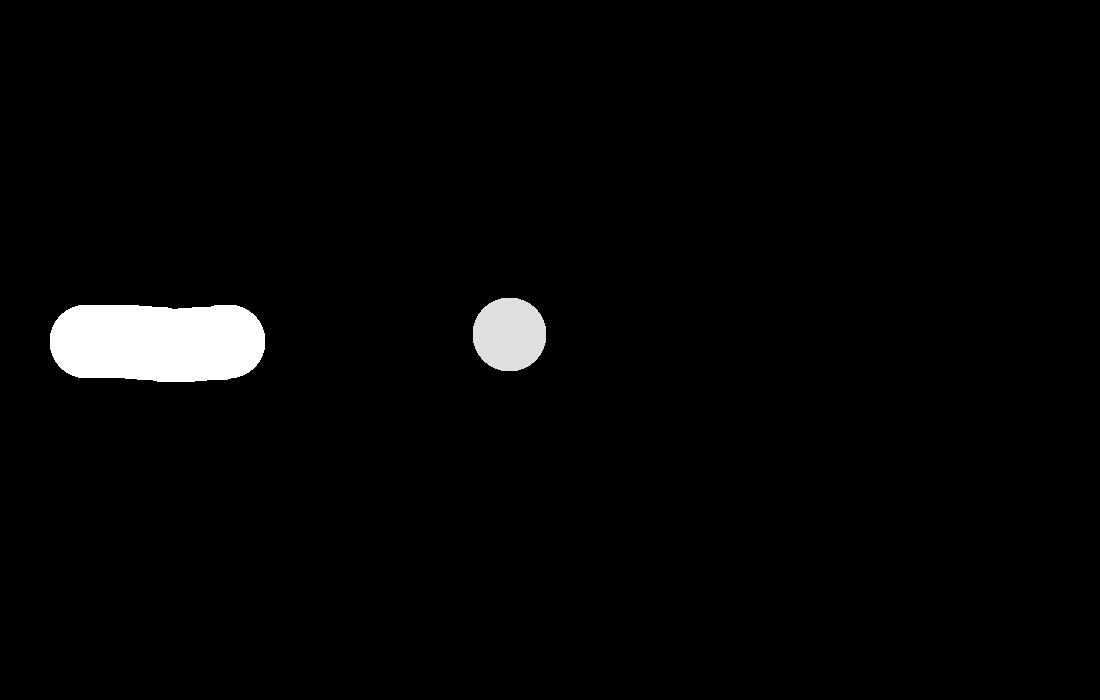

Supplement: S1 Software — This package contains a routine for computing the AOGM measure. (ZIP) [file pone.0144959.s001.zip › testing_data/RES/mask005.tif]

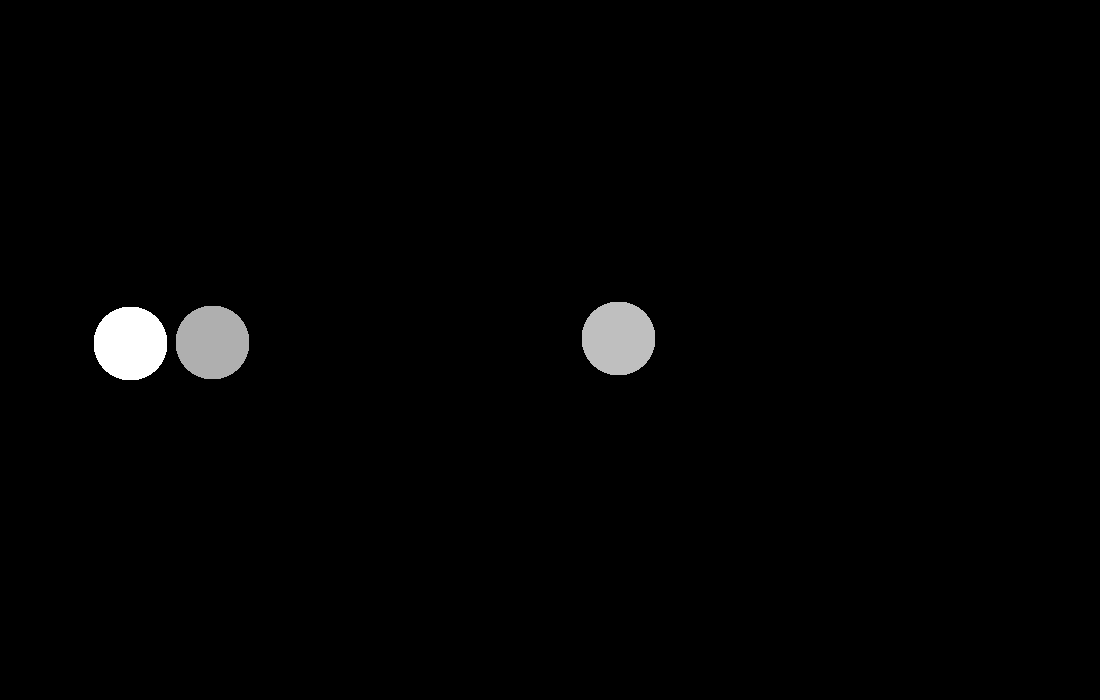

Supplement: S1 Software — This package contains a routine for computing the AOGM measure. (ZIP) [file pone.0144959.s001.zip › testing_data/RES/mask006.tif]
